# Extra Supplement—The Nursing Mirror

**Published:** 1889-09-28

**Authors:** 


*Extra Supplement*

### The Nursing Mirror.

All Contributions for this Supplement should be addressed to the Editor, The Hospital, 139-140, Salisbury Court, Fleet Street, London, E.C., and should have the word "Nursing" plainly written in left-hand top corner of the envelope

### En Passant.

**News from the East.**—Mrs. Bishop (Miss Bird), who is travelling in the East, has written home good news of her hospital projects. Before Mrs. Bishop left Kashmir, the Maharajah made a grant of a very eligible piece of land in Srinagar, on which she is building a completely equipped women's dispensary and a women's hospital of 60 beds, to be called "The John Bishop Memorial Hospital." Both these buildings will be under the charge of Miss Butler, M.D., with assistant and a trained nurse sent out by the Church of England Zenana Missionary Society.

**Marriage of a Matron.**—Last week's *Graphic* contained a half-page illustration of the marriage of Miss S. E. Elcock, late matron of the Jaffray Hospital, Birmingham, and now Mrs. Rhead. The wedding was eminently picturesque, for the six bridesmaids were all nurses from the hospital, and were all dressed in uniform, including the neat caps, with strings tying under the chin. They all carried exquisite baskets of flowers. Mr. Jaffray, the founder of the hospital, gave away the bride, for whose labours he has the greatest admiration. The wedding was solemnised on the 7th inst. at Erdington parish church by the Rev. Canon Bowlby and the vicar.

**The Doll Competition.**—The following questions and answers clears up one or two points: E. T. W. asks: "Will it be allowable to have the doll's caps and aprons washed and ironed?" [Certainly.] H. L. A. asks: "What is to become of the dolls? Would it not be possible to keep them six or twelve months to enable country nurses who cannot get away now to see them?" [This question was answered in The Mirror of the 14th inst. Arrangements will be made as far as possible to give everybody an opportunity of seeing them.] Sister Margery asks: "Would it be fair to compete for the doll exhibition if the clothes were made by one of her patients under her supervision, as she would not have time to make all herself." [Anyone so competing must state by whom the work has been done, or they will be otherwise disqualified.]

**Attendants on the Insane.**—The subject of the use of mechanical restraint in acute cases, has lately been brought forward in regard to a case at Bethlem Hospital. A lady subject to mania was put under restraint at home, but was finally sent to the asylum where bodily restraint is forbidden. A writer to the *Midland Times* says: "It would be far better, in my opinion, if they did use reasonable and proper restraint when required, as the lady in question, in a letter to her parents, said she was filled with horror at being put in a dark cell one night, and could never forget it, 'not if she lived for an eternity of ages.' On the other hand, when asked by the doctor to show him how the restraint at her own home was used, answered, 'with pleasure,' and proceeded at once to show him. Any unprejudiced person would, I should think, say without hesitation that it was far more cruel to cause a patient suffering from such a distressing complaint to be heavily drugged, or held day and night by four or five nurses, as required, or placed in a dark cell, than to be restrained, when necessary, in the manner indicated. Nurses have tempers like other people, and doctors cannot be always with their patients."

**Short Items.**—Seven Birmingham nurses are down with scarlet fever.—Miss Singleton, the matron of the Convalescent Home at Parkgate, near Chester, received recently a visit from Mrs. Gladstone, who went all over the building, conversed with the inmates, and expressed herself much pleased with all the arrangements. Mrs. Gladstone on leaving promised to bring Mr. Gladstone with her on her next visit.—Miss Kate Murphy, who is shortly going out to India for zenana and medical work in the Mesbudda Valley, took leave of her well-wishers at the Friends' Mission Hall, Oxford, on the 10th inst.—Miss Close has recovered from her indisposition, and has now commenced her duties at the Royal Infirmary, Newcastle-on-Tyne.—Five nuns of the Third Order of St. Dominic lately arrived at Cuenca, Ecuador, to take charge of a leper settlement there. The same sisters also conduct a large hospital for lepers in Trinidad.

**Puerperal Fever.**—All midwives ought to study the evidence given at the inquest on Eleanor Bamforth last week. Mrs. Margaret Lyddon, in reply to the coroner, said that, although uncertificated, both she and her sister acted as midwives, having learnt the business from their mother. On August 5 an inquest was held on a woman named Ward, who died of puerperal fever. The coroner warned witness that the disease was contagious, and that as she had atended Ward she ought not to attend any other confinements for a fortnight. Pressed by the coroner, witness admitted that she attended a woman in Church-row, who also died of puerperal fever, but that was before the inquest referred to. On the 14th of August she called to a woman in Salter-street. She went, but told the woman she ought not to attend, but the woman replied that she was not nervous, as people had to die when their time came and not before. Witness performed that confinement, and the woman died of the same complaint. She attended another woman named Perriott at 2, Oak-lane, who also died in a similar way. On the 24th of July she attended Mrs. Sparrow, at 65, Copenhagen-place. This woman died, and Dr. Linton was called in. He told witness that she ought not to go out for a fortnight. Mrs. Lyddon, however, attended her own daughter, Eleanor Bamforth, through her confinement, and the daughter died of puerperal fever; the baby died also. Dr. Russell Maine Talbot, medical officer of health, deposed to the cause of her death. The Coroner: If a woman attends a case where puerperal fever exists, can she convey it to another case? Witness: Oh, yes; and it is difficult to say how long she should abstain from attending confinements, for it is conveyed in the clothes of persons, and even in their breath. The Coroner: How would you suggest that it was conveyed? Witness: Well, Mrs. Lyddon is herself suffering from a constitutional disorder and I think the first case was set up by that. He had attended her for a piece of dead bone in the mouth, and if she had been fingering it that might account for the outbreak, as it would probably set the contagious epidemic going. He was of opinion that nothing less than three months' quarantine would clear Mrs. Lyddon and the prospect of further spreading the disease. The case is too clear and too terrible to need comment; here are seven deaths attributable to the carelessness of a so-called midwife. We cannot but be thankful that the Midwives' Institute has introduced a Bill into Parliament for the compulsory registration of midwives.

### Case Taking.

FIRST PAPER.

It often happens to a nurse trained in a large hospital, and then sent to work in some country infirmary or cottage hospital, to have to take cases, though she has never been instructed in this work. Where there are plenty of students and dressers about, as well as house surgeons and physicians, there is no necessity for a nurse to keep a case-book, though it would be wiser for her to do so. There are a few schools—St. Thomas's, for instance—where case-taking is taught to nurses; but, on the contrary, there are hospitals where, if a nurse is seen reading the notes of dressers or physicians, she is sharply bidden to mind her own business. Truly, a nurse's work is so great and tiring that unless she is encouraged to do so, she seldom takes an intelligent view of the treatment carried on in her ward; and then, when she goes into rural districts where her responsibilities are great, and students there are none, she is rather at a loss to know how to get on. There are the familiar rows of beds, there are the patients, but where are the entrance tickets, the prescription and case boards? "What is the age of this man, nurse?" asks the medical officer. The nurse's eye glances above the bed, but no information hangs there. She searches in her memory, but has very naturally forgotten the small fact. How much better had she been able to draw a neat little case-book from her pocket, and turning to a numbered page been able to see at a glance every particular of the case the doctor could care to know? It is all very well to say that infirmaries and cottage hospitals are dreary places, where little can be learnt, but a nurse ought to be something more than a child who requires constant drilling—she ought to be able to teach herself. Then she will find that every case she nurses is full of interest and full of instruction.

Case-taking consists in the systematic keeping of written notes on the history, state, appearance, and treatment of a patient. The first thing to discover is the patient's name, age, and occupation, and with these should be noted the sex and date of admission. Then when the patient has been put to bed, and has had a meal, it is well to question both him and any friends who may have brought him as to (1) his personal history, (2) his family history, (3) the history of his present illness. With regard to personal history, the chief points to discover are notes of previous illnesses, and what the man's habits of life have been. Every nurse must know how cases of heart disease can be traced back to an attack of rheumatic fever, or cases of Bright's disease to an attack of scarlet fever. When the accounts of a previous illness differ as told by the patient, and as told by the friend, it is always well to note from whom the statement was taken; begin "Patient says," or "Patient's brother says." The reason for noting whether the patient has been in the habit of drinking, whether his occupation is an unhealthy one, whether he has lived a fast life, are obvious; all these things may throw light on his present state. The family history is necessary for tracing causes for hereditary disease; it should include special enquiries as to consumption, cancer, gout, fits, insanity, etc., in any of patients' relations; and should state whether parents, and brothers, and sisters, are alive, and if not, of what they died and at what age. The history of the present illness includes the date when the patient first felt unwell and his first symptoms. His daily symptoms from then till he came to the ward should be noted; special enquiries being made as to what medicines were taken, and at whose direction; what food was eaten; whether there was any sickness, etc.

*(To be continued.)*

### A Probationer's Diary.

*(Continued from page lxxiii.)*

*Friday, Oct. 26.*—A new probationer from our Birmingham Nursing Institution, to which I am very proud to belong, arrived to-day. This hospital has the privilege of training six every year; as the one completes her training and returns to the home, for private nursing, another takes her place here. However, to the routine: our ovarian most satisfactory, ice and milk, 1oz. every hour. Two new patients, two girls, both chorea: bath each and to bed. A very heavy fog, gas all over hospital greater part of day.

*Saturday.*—The linen belonging to surgical wards is kept in the nurses' day or dining-room, and all brought up from laundry and put on the tables to be stored by the nurse on Saturday, so that the nurses on this above all other days do not belong to the unemployed. Our ovarian progressing; both chorea cases improving.

*Sunday.*—Three of our honorary surgeons visited the wards this morning. On duty by myself. Went to the little hospital chapel. The visiting hour seems to pass all too quickly for the visitors. It is a lesson never to be passed over—the anxious, pleased look on the faces of all as their own kith and kin walk into the wards. It is "love," implanted by God Almighty, for one's own in every path of life, and in ill-health it shines the best.

*Monday.*—I've been here a month to-day. A new patient, a poor woman, who has crushed her middle finger, punished herself by trying her home cures, failing, comes here. It is very nasty to look at, and has a disagreeable odour. Bath and to bed. The assistant house surgeon administered an anæsthetic to her, and when she was nearly unconscious discovered her top teeth were artificial. Luckily got them out. The house surgeon quickly amputated to first joint, applied torsion to the bloodvessels, folded a neat piece of flesh from either side over top, and put a dressing on of carbolised gauze. Patient very soon was herself, very little sick, and though in hospital but a short time, very grateful. Went to a lecture in the evening in Bell Library. Principally about fractures, and the skull being composed of three layers—the outer smooth and hard, the middle spongy or porous, and the inner smooth, like the outer. Also surrounded by three things: first, a membrane (like the periosteum); secondly, a watery fluid; and thirdly, next the brain a thin membrane. Our skull in some places is half an inch thick, while in others it is about the sixth of an inch.

*Tuesday.*—I learned, while at dinner to-day, that the man who was confined so long in a coal mine is going on very well since the amputation of his leg. The other leg has quite recovered its circulation and gives no pain. Our ovarian tumour patient is allowed 1½oz. in the hour, and her temperature is normal. The wound dressed for the second time to-day, and four stitches removed. A new patient, a poor woman, with tumour in arm. The ovarian still has a special nurse for night duty.

*Wednesday.*—The children's ward being full, we have a child sent here, almost covered with boils. The poor little girl cried as we got her into a warm bath, but soon the water comforted her. I could not get her really clean, though I used a little linseed oil. Put her to bed in a blanket, made small poultices, and strapped them on. Four convalescent patients dismissed.

*Thursday.*—Fearful fight outside the hospital; some boot-makers on strike. Sundry nasty wounds to be dressed in receiving-room. Our usual routine; nothing fresh. I went to Birmingham, and arrived back at hospital at 9.30, awfully tired in body, but refreshed in mind, and ready for to-morrow's work.

*Friday.*—Glorious autumn morning; all our patients mending; one chorea girl gone out, the child with boils more comfortable, and enjoys her two warm baths daily. Another girl had a bad knee asperated.

*Saturday.*—I went over to the smallpox wards to a man about forty. They are not nice wards; I did not mind the day's work, and am to go back to main block to-night. I hear a new smallpox ward is to be built.

*Sunday.*—A quiet day with my smallpox patient.

*Monday.*—After breakfast I came to hospital, but did not go on duty till afternoon, then in men's accident ward. Two accidents admitted, one arm torn badly in machinery, torsion to the smaller bloodvessels, ligature to the larger, and arm bound up on splint. Second case, an engine-driver, three toes crushed; ether, the crushed parts quickly removed, foot bandaged, and patient put to bed. Very trying day, so I managed to close it with a fearful headache; could not sleep for hours.

*Tuesday.*—I hear that last evening the accidents in the receiving-room were very few. No accidents to-day; the two patients admitted yesterday going on favourably.

*Wednesday.*—Our lecture to-day, instead of Monday. A long letter from home; how such trifles give zest to the work! A little boy admitted with broken tibia; run over. A bad burn case on the 5th, from fireworks. Admitted a little girl with fractured skull and tibia—also run over; attended to in receiving-room, and carried to children's ward, followed by her heart-broken mother.

*Thursday.*—One of our probationers ill with scarlet fever taken to the fever block. I hear the little girl admitted yesterday has passed into a better keeping than ours. Our little laddie bright, sitting up; his leg does not seem to trouble him. Our burnt case improving. The man with arm torn in machinery mending; but the house surgeon made two small incisions where it looked angry.

### Doll Competition.

The following forty institutions will be represented at the Doll Show, and we invite nurses belonging to other institutions to enter their names before Oct. 1, and so make the show more complete:

Bucks. Infirmary.
Cheltenham Nursing Institution.
Cork Children's Hospital.
Deaconesses' Institution, Tottenham.
Devon and Exeter Hospital.
Derby Children's Hospital.
Fir's Home, Bournemouth.
Glasgow Sick Poor Association.
Glasgow Western Infirmary.
Glasgow Hospital for Children.
Grimsby and District Hospital.
Home Hospital, Weymouth-street.
Home Hospital, Fitzroy House.
Home Hospital, Hampstead.
Hahnemann Hospital, Liverpool.
Isolation Hospital, Tatterdown-lane.
King's College Hospital.
London Hospital.
London Fever Hospital.
Leeds Nurses' Institution.
Leicester Infirmary.
Northampton Nursing Institution.
National Hospital, Queen's-square.
Pendlebury Children's Hospital.
Private Home, Portland-place.
Royal Berks. Hospital.
Radcliffe Infirmary.
Sussex County Hospital.
South Devon Hospital.
Sheffield Children's Hospital.
St. Marylebone Infirmary.
Stamford Nursing Society.
St. Mary's Hospital, Paddington.
St. Helen's Institute, Jersey.
St. John the Evangelist Institute, Strand.
St. Thomas's Hospital.
Truro Infirmary.
West Malling Private Institute.
Wandsworth and Clapham Infirmary.
Wilkington Hospital, Manchester.

### Everybody's Opinion.

*[Correspondence on all subjects is invited, but we cannot in any way be responsible for the opinions expressed by our correspondents. No communications can be entertained if the name and address of the correspondent is not given, or unless one side of the paper only be written on.]*

### COMBINATION v. INSURANCE.

A Hospital Life Governor writes: "Nurse E.'s" position as described in your last week's number is entitled to much commiseration. Especially when it is notorious that the nursing institutions and hospitals are making large profits out of the earnings of the nurses without apportioning to them any adequate superannuation allowances in return for services faithfully rendered. Surely after encountering the many dangers incidental to a nurse's calling, "Nurse E." should at the age named (43) have had some reasonable provision to meet her necessary wants reserved by her employers. But why cannot the qualified nurses, both seniors and juniors, combine upon some principle of mutual assurance, based upon a regulation commanding the confidence of the public, and themselves carry on the practice of private nursing? Possibly the National Pension Fund Society might make some arrangement that would tend to relieve those who, like "Nurse E.," are exposed to such unmerited misfortune. There are many of them, and they are entitled from every point of view, from the nature of the conscientious and anxious duties they undertake, to the greatest consideration on the part of the public.

### NURSING THE INSANE.

A Constant Reader writes: In the Nursing Mirror of August 14 you ask for our experience in managing the insane. For nearly twenty years I have made insanity my constant study, and for nearly as many years have had the daily care of patients. I have been lady superintendent of three asylums, and for the last few years have had the care of private cases given me by different London doctors. My experience has taught me that if friends have means the patient is better out of an asylum. Patients herded together learn tricks one from each other, and after recovery do not forget asylum life. All experienced people will own that patients are better managed away from friends. Patients themselves are very difficult to manage and get on with, but their friends are much more so. With regard to nurses managing a single patient or group best, it depends entirely upon the nurse. I have nurses who make capital ward attendants, but failed to get on with a single case, and *vice versa*. At this present time I have a lady patient given me by a well-known doctor. She has been under my care for the last two years (it is a homicidal case). During the two years one has been spent in travelling in different parts of England, the Continent, and Northern Africa, and one year in the same house as her parents. I need hardly tell you that my charge was much better away from home, and more easily managed. At home, if left to herself, she would soon go back into her old habits and tricks. I quite dread seeing any members of her family come near us.

### SUPERANNUATED AT FORTY-THREE.

M. E. Smith writes: Allow me to state my case, which is similar to that of "Nurse E.," the writer of the letter in this week's Hospital. She is one of the first thousand. I was most anxious to be one of them too, but found myself unable, because, being a little over forty, I am considered getting too old for nursing. Although still very active, I must confess that I am not so able to work night and day as I used to be, nor to do hard night nursing for months together as I have many times done. Still, I feel there is at least ten years' more work in me. I find that I have done sixteen years' nursing altogether, ten of which have been in private families. My hospital work was as follows: Manchester Royal Infirmary, fourteen months; Guy's Hospital, twenty-one months; two unions in infectious wards, eighteen months; asylums, county and private, fourteen months; lying-in hospitals, five months. I hold Obstetrical Society's diploma. I should be glad to show my many testimonials, hospital and from private patients. It is not a pension just yet, but suitable occupation at a reasonable remuneration, that is wanted for many like "Nurse E." and myself; who, having given their services to the sick when they required them, would like to feel that they were in their turn not left out in the cold as they get older.

### NORFOLK AND NORWICH HOSPITAL.

MR. HOWARD J. COLLINS writes: Will you kindly allow me to correct an error in the article on "How to become a Nurse" in last week's issue. Therein it is stated that "at Norwich and elsewhere in the country a certificate is given at the end of a year," whereas probationers are only received here on the following conditions. Probationers come for a month on trial, and, if willing and found suitable, are then required to sign an agreement to serve the hospital for *three* years, during which time lectures are delivered to them by members of the honorary medical staff in elementary anatomy and physiology and in medical and surgical nursing, as well as the practical instruction given them by the head nurse of the ward. At the end of that time they are required to pass an examination to the satisfaction of a physician and surgeon of the hospital, and if well reported of by the lady superintendent are granted certificates, signed by the above two members of the honorary medical staff and the lady superintendent, and countersigned by the board of management.

[Another correspondent points out that at St. Mary's, Paddington, a paying probationer receives a certificate at the end of one year.—ED. MIRROR.]

### A NURSE'S TRIALS.

DORIS writes: I do not think "Student H." would find my nails dirty or badly trimmed. I try to make the most of them, as my hands are otherwise ugly. I wear them rather short, barely to the tips of my fingers. I should, perhaps, say the idea is not my own. I read it first in the *Girl's Own Paper*, I think, and afterwards heard it referred to in a lecture on anatomy.

### ASYLUM ATTENDANT.

EX-ASYLUM NURSE writes: I must ask you to insert the following contradiction to a letter from "An Asylum Attendant" appearing in your issue of this week. For five years I was an asylum nurse in one of the largest asylums in Lancashire, and the medical superintendent constantly gave the nurses and male attendants both lectures and instruction. We had lectures both on an insane mind and a sane mind, also instruction in bandaging, etc., etc. "An Asylum Attendant" states that many nurses are injured by patients. To my idea the asylum that he has had experience in must either be very small or badly managed, by there being few nurses; as generally, in the event of an attack being made, there is plenty of help at hand. During the whole time I was a nurse no one in the asylum was injured, and the nurses and attendants always received the greatest consideration from the superintendent, junior doctors, and matron, and they all tried to give us every help and instruction; therefore we had neither to work in the dark or guess at our duties.

### Appointments.

**[It is requested that successful candidates will send a copy of their applications and testimonials, with date of election, to THE EDITOR, The Lodge, Porchester-square, W.]**

DOSHISHA HOSPITAL.—Miss Richards, formerly of the Boston Training School, has been appointed superintendent of the first Christian training school for nurses in Japan in connection with the Doshisha Hospital. This training school and hospital will be the basis of a contemplated medical school in connection with Doshisha College.

### Vacancies.

**The following vacancies are announced:**

**Matron.**

Epilepsy and Paralysis Hospital, £50.

Salisbury Infirmary, £75.

York Lunatic Asylum, £60.

**Night Superintendent.**

Royal Portsmouth Hospital.

**Sisters.**

Bolton Infirmary.
Bradford Infectious Hospital, £35

**Nurses.**

Abroath Infirmary, £20.

Barnstaple Infirmary.

Bexley Cottage Hospital.

Birmingham Eye Hospital (assistant), £15.

Bradford Infectious Hospital, £23.

Brecon County Infirmary, £18.

Brighton Fever Hospital, £28.

Bury Dispensary Hospital, £25.

Bury Hospital for Incurables (night)

Canterbury Hospital (night), £22.

Carmarthen Infirmary, £23.

Cheltenham Nursing Institution.

Chester Diocesan Deaconess' Institution (several), £24.

Chester General Infirmary (fever).

Eccles and Patricroft Hospital, £20.

Frome Nurses' Home, £20.

Heart Hospital, Soho-square.

Highbury Institute (several), £20.

Isle of Wight Infirmary, £22.

Kent Nursing Institution.

Lock Hospital, Harrow-road.

Morley's Convalescent Hospital, £23

Northern Fever Hospital, £30.

Norwood Cottage Hospital.

Nurses' Home, Hull, £25.

Nurses' Institution, York.

Nursing Institution, 96, 97, 98 Wimpole-street, London, W., Mr. Wilson has several vacancies for thoroughly trained nurses.

Peterborough Infirmary.

River's Street Home, Bath.

Scarborough Home Hospital.

Sheffield Nurses' Home.

St. John's Institution, Southport.

Victoria Home, Bournemouth.

West Derby Union Infirmary, £25

Weston-super-Mare Hospital (night)

Wolverhampton Victoria Institution.

**Probationers.**

Grimsby Hospital.
Horton Infirmary.
Sheffield Nurses' Home.
Taunton and Somerset Hospital.
Weston-super-Mare Hospital.
Workhouse Infirmary Nursing Association.
Yarrow Memorial Hospital

### Notes and Queries.

NOTICE.—We must really ask our correspondents to be more particular in enclosing name and address. Constantly we receive queries or letters with no name attached.

**Queries.**

*Nurse E.*—Will Nurse E. kindly send her address to the editor, as letter has been sent to THE HOSPITAL office for her.

(93) *Beef Tea.*—Nurse Davies will be glad to know if "beef tea," made as recommended by Dr. Moore Jessops, would be given to patients suffering from pleurisy or tubercular peritonitis, or where there was any tendency to indigestion, as she has nursed cases where the doctor has been much against "grownds" in beef tea and chicken tea.

(94) What is Addison's disease? *Private Nurse.*

**Answers.**

*Daisy.*—The best way to get an appointment in South Africa is to watch our advertisement columns.

(79) *Lady Roberts' Homes.*—All information concerning these homes can be found on page 163, Vol. III. of THE HOSPITAL, where there is an article by Lady Roberts. The scheme is in no way connected with Lady Dufferin's Fund, and is for supplying homes in the hills where nurses can be moved during the hot season. Such a home is open at Murree. This query has been constantly answered before. We wish our correspondents would watch this column. *Editor.*

(80) *Red Cross Nurses.*—The last volume of the *Sunday at Home* contains the information "K. B." requires. Perhaps Mrs. Brewer, who wrote the articles, would answer privately if "K. B." addressed her at the office of the *Sunday at Home*, 56, Paternoster-row.

(83) *Case-Book.*—Dr. Jeaffreson, of Newcastle-on-Tyne, has compiled a "Nursing Chart," which offers an easy and simple method of recording what a patient has taken in the way of medicine, food, stimulants, etc., and the effects of these upon the system generally, and the secretions. Wright, of Bristol, also publishes pocket charts for noting all points of a case a nurse ought to remember. Fifty charts cost 2s. 6d.

(85) *Second-Hand Medical Books.*—Apply to Kimpton's, booksellers High Holborn.

(86) *Cramp.*—It is impossible for us to prescribe for cramp, or to say whether massage would benefit a patient we have never seen.

(87) *Dispensing.*—Apply at the Zenana Medical College, 58, St. George's road, S.W., or to Dr. Annie McCall, 131, Clapham-road, or to the New Hospital for Women. This query has been constantly answered before.

(88) *Books on Monthly Nursing.*—See answers to query (71) in our numbers for September 7th and August 30th.

(89) *Dilemma* should read the article "How to Become a Nurse" in our number for August 30th. Hoblyn's is the cheapest and best Dictionary of Medical Terms.

(90) *Training Schools.*—Apply to the Matron of Chichester Infirmary. Watch our advertisement columns.

(91) Our answer to *White Face's* query is again that we never prescribe or venture to remark on the health of a person we have never seen.

(92) *Training Schools.*—Read the article "How to become a Nurse" in our number for August 30th. Three months' training in a lying-in hospital should prepare you to take the certificate of the London Obstetrical Society.

